# A Study on the Correlations Between Comorbid Disease Conditions and Central and Peripheral Neurological Manifestations of COVID-19

**DOI:** 10.7759/cureus.29838

**Published:** 2022-10-02

**Authors:** Anya Vaish, Sudeshna Ray, Brad Tyson

**Affiliations:** 1 Neurology, Tesla Stem High School, Redmond, USA; 2 Emergency Medicine, Eastside Research, Redmond, USA; 3 Neuropsychology, Evergreen Neuroscience Institute, EvergreenHealth Medical Center, Kirkland, USA

**Keywords:** comorbid conditions, neurological manifestations, cardiovascular diseases, cns, covid-19

## Abstract

Background

Medical comorbidities and neurological manifestations are commonly associated with COVID-19, though specific relationships remain unclear.

Objective

The aim of this study is to investigate the relationship between medical comorbidities and neurological manifestations in patients with COVID-19.

Methods

We reviewed medical comorbidities and COVID-19-related central nervous system (CNS) and peripheral nervous system (PNS) manifestations in 484 consecutive patients with COVID-19.

Results

Neurological manifestations were seen in 345 (71%) of 484 COVID-19 patients. CNS manifestations included headaches (22%), altered mental status (19%), dizziness (8%), gait imbalance (5%), strokes (four patients, <1%), and seizures (two patients, <1%). PNS manifestations included myalgia (31%), hypogeusia (8%), hyposmia (6%), critical illness myopathy (nine patients, 2%), visual disturbance (six patients, 1%), rhabdomyolysis (four patients, <1%), and nerve pain (one patient, <1%). There were 153 (32%) patients with CNS manifestations, 98 (20%) patients with PNS manifestations, and 94 (19%) patients with combined CNS and PNS manifestations. Comorbidities such as cardiac disease (22%), dementia (17%), hypertension (16%), and chronic obstructive pulmonary disease (COPD; 13%) were significantly associated with CNS manifestations. No comorbidities were associated with PNS manifestations.

Conclusion

Neurological manifestations were common in our sample of 484 COVID-19 patients, with headache and altered mental status being the most common CNS manifestations and myalgia being the most common PNS manifestation. Cardiac disease, dementia, hypertension, and COPD were more common in patients with CNS manifestations. Providers should be vigilant about the possible emergence of CNS manifestations in COVID-19 patients with these comorbid conditions.

## Introduction

The severe acute respiratory syndrome coronavirus 2 (SARS-CoV-2) and the disease it causes, coronavirus disease 2019 (COVID-19), can present with a range of manifestations such as fever, cough, shortness of breath, fatigue, nausea, vomiting, and diarrhea. Multiple medical comorbidities have been linked to severe disease and mortality, including cardiovascular diseases, cancer, chronic kidney disease, chronic lung diseases, dementia, diabetes mellitus, and obesity. Research has also documented a diverse constellation of central nervous system (CNS) and peripheral nervous system (PNS) manifestations including altered mental status, dizziness, gait imbalance, headache, hyposmia, hypogeusia, seizure, and stroke [1-3]. These neurological signs and symptoms have been reported in more than half of hospitalized patients with COVID-19 and are associated with an increased risk of mortality [1,4,5]. Interestingly, involvement of the CNS and PNS is independent of the severity of the respiratory disease, presenting a challenge for treating neurologists.

While medical comorbidities and neurological manifestations have well-documented associations with COVID-19, there remains much to learn about the specific nature of these relationships. As such, it is important to understand which medical comorbidities increase the risk of neurological manifestations in patients with COVID-19 and whether specific comorbidities increase the risk for CNS or PNS manifestations. This study investigates the association between medical comorbidities and CNS and PNS manifestations in patients with COVID-19 to further elucidate the nature of these relationships.

## Materials and methods

Participants

Data were collected through a clinical chart review of 484 consecutive patients with SARS-CoV-2 infection seen in outpatient clinics and the hospital between February 20, 2020, and July 4, 2020, at EvergreenHealth Medical Center in Kirkland, WA, the first hospital with reported cases in the United States. All patients had SARS-CoV-2 infection confirmed by polymerase chain reaction testing of a nasopharyngeal sample. The study was approved by Western Institutional Review Board, which is our institutional review board and ethics committee.

Materials

Data included demographic characteristics, medical history, and presenting manifestations. Only new-onset neurological manifestations were analyzed. Chart notes were independently reviewed by a neurologist and neuropsychologist, and data were cross-referenced for accuracy. Presenting signs and symptoms were reported by patients, family members, care partners, nursing staff, emergency responders, and physicians.

Statistical analysis

Descriptive statistics (mean [*M*], standard deviation [*SD*], percentage) were computed for relevant variables. Categorical variables were presented as absolute values along with percentages and compared using the Pearson *χ*^2^ test. All tests were two-sided, with a p-value less than 0.05 considered statistically significant. All 345 patients displaying neurological signs and symptoms were divided into three groups: (1) patients with only CNS manifestations, (2) patients with only PNS manifestations, and (3) patients with both CNS and PNS manifestations. Chi-square tests were carried out using Excel functions and null hypothesis. The critical *χ*^2^ value for comparison of three groups was calculated using two degrees of freedom with a p-value of 0.05. The problem *χ*^2^ was calculated for each comorbid condition by summing the values calculated using the formula (O-E)^2^/E for each CNS, PNS, and CNS/PNS groups, where O = observed frequency of CNS, PNS, or CNS/PNS group, and E = expected frequency of the comorbid condition of each of the CNS, PNS, or CNS/PNS group. If the problem *χ*^2^ value for the comorbid condition is greater than the calculated *χ*^2^ value of the comorbid condition group, the null hypothesis was rejected, indicating that the difference between observed frequencies and expected frequencies is large enough to be considered statistically significant.

## Results

Clinical characteristics and preadmission comorbidities of patients with nervous system involvement are presented in Table 1. Neurological manifestations were seen in 345 (71%) of 484 COVID-19 patients. The average age of patients with neurological manifestations was 59 years (*M* = 58.8, *SD* = 20.6). The majority were Caucasian/white (79.4%), with slightly more females (51%). In our sample of 484 COVID-19 patients, there were 153 (32%) patients with CNS manifestations, 98 (20%) patients with PNS manifestations, and 94 (19%) patients with combined CNS and PNS manifestations. CNS manifestations included headaches (107 patients, 22%), altered mental status (92 patients, 19%), dizziness (40 patients, 8%), gait imbalance (23 patients, 5%), strokes (four patients, <1%), and seizures (two patients, <1%). PNS manifestations included myalgia (151 patients, 31%), hypogeusia (38 patients, 8%), hyposmia (27 patients, 6%), critical illness myopathy (nine patients, 2%), visual disturbance (six patients, 1%), rhabdomyolysis (four patients, <1%), and nerve pain (one patient, <1%). The most common comorbid condition associated with CNS manifestations was cardiac disease. Comorbidities such as cardiac disease (108 patients, 22%), dementia (83 patients, 17%), hypertension (76 patients, 16%), and COPD (61 patients, 13%) were significantly associated with CNS manifestations. No comorbidities were associated with PNS manifestations.

**Table 1:** Clinical characteristics and preadmission comorbidities of all 345 COVID-19 patients displaying CNS and/or PNS symptoms. Percentages were calculated based on 345 patients displaying preadmission comorbidities. Critical chi-square value of 5.991464 is based on p = 0.05 and degree of freedom (df) = 2. †The numbers are too small for meaningful chi-square comparison. COPD, chronic obstructive pulmonary disease

	CNS only symptoms (153 patients)	PNS only symptoms (98 patients)	CNS and PNS symptoms (94 patients)	Calculated chi-square value from the given problem
Characteristics	No. (%) of patients	No. (%) of patients	No. (%) of patients	
Age, years				
≥65	108 (31.3)	16 (4.6)	16 (4.6)	
<65	45 (13.0)	82 (23.8)	78 (22.6)	
Gender				
Male	73 (21.2)	43 (12.5)	53 (15.4)	
Female	80 (23.2)	55 (15.9)	41 (11.9)	
Race				
Caucasian	127 (36.8)	75 (21.7)	72 (20.9)	
African American	3 (0.9)	3 (0.9)	3 (0.9)	
Asian	16 (4.6)	8 (2.3)	12 (3.5)	
Native Hawaiian or Pacific Islander	1 (0.3)	4 (1.2)	0 (0.0)	
American Indian or Alaska Native	0 (0.0)	1 (0.3)	2 (0.6)	
Undetermined	6 (1.7)	7 (2.0)	5 (1.4)	
Core symptoms				
Fever	95 (27.5)	69 (20.0)	54 (15.7)	
Cough	95 (27.5)	71 (20.6)	73 (21.2)	
Shortness of breath	75 (21.7)	49 (14.2)	52 (15.1)	
Preadmission comorbidities				
Cardiovascular disease	72 (20.9)	14 (4.1)	22 (6.4)	54.89
Peripheral vascular disease	12 (3.5)	13 (3.8)	5 (1.4)	3.80
Dementia	64 (18.6)	6 (1.7)	13 (3.8)	72.46
COPD	34 (9.9)	17 (4.9)	10 (2.9)	14.98
Diabetes mellitus	43 (12.5)	23 (6.7)	21 (6.1)	10.21
Chronic kidney disease	37 (10.7)	4(1.2)	12 3.5()	33.55
Asthma^†^	11 (3.2)	9 (2.6)	2 (0.6)	6.09
Cancer^†^	10 (2.9)	2 (0.6)	2 (0.6)	9.14
Hypertension	50 (14.5)	10 (2.9)	16 (4.6)	36.73
Rheumatoid arthritis^†^	9 (2.6)	1 (0.3)	2 (0.6)	9.50
Others	22 (6.4)	7 (2.0)	6 (1.7)	13.77

## Discussion

In our review of 484 patients with COVID-19 presenting to outpatient clinics and the emergency department, 345 (71%) had neurological manifestations. Of the 345 patients with neurological manifestations, 153 (32%) had CNS manifestations, 98 (20%) had PNS manifestations, and 94 (19%) had both CNS and PNS manifestations. Several comorbid conditions were associated with CNS manifestations (cardiac disease, dementia, hypertension, and COPD), though no comorbidities were associated with PNS manifestations. Our results are largely consistent with those of prior studies and suggest that neurological signs and symptoms are common presenting features of COVID-19 [6-8].

The pathophysiology of neurological manifestations in COVID-19 is mechanistically diverse and includes direct neuroinvasion, immune dysregulation and systemic inflammation, hypoxic-ischemic processes, endothelial damage and microvascular injury, maladaptation of the angiotensin-converting enzyme (ACE2) pathway, and the unique psychosocial impacts of this infection and related pandemic [5,9]. Several studies have reported the presence of SARS-CoV-2 in the cerebral spinal fluid and postmortem brain tissue of COVID-19 patients with encephalitis [10,11]. Early reports suggested that SARS-CoV-2 may gain access through nasal epithelial cells, infiltrating the bloodstream and lymph to reach other tissues [12-15]. However, while the neurotrophic properties of the virus represent one potential route to neurological dysfunction, research indicates that neurological complications are more commonly the result of severe systemic inflammation rather than direct neuroinvasion [16]. Immune-mediated mechanisms influence function of macrophages, microglia, and astrocytes, and are closely related to the development of a systemic inflammatory response. The neurovirulence of COVID-19 correlates with its ability to induce proinflammatory cytokine signals from astrocytes and microglia. SARS-CoV-2 can promote a proinflammatory state by activating glial cells [17]. Virus proliferation in lung tissue may precipitate cerebral hypoxia and anaerobic metabolism, leading to manifestations such as altered mental status, dizziness, gait imbalance, and stroke. This is particularly true for vulnerable individuals such as those with cardiac disease, hypertension, dementia, and COPD [4]. Strokes have been documented extensively in COVID-19 patients, though less than 1% of our patients experienced stroke.

Early research demonstrated that SARS-CoV-2 attaches to ACE2 receptors in the capillary endothelium [18,19], which, in turn, may cause abnormally elevated blood pressure, acute cerebral infarction or hemorrhage, and/or cerebral sinus venous thrombosis. ACE2 is expressed in various organs including the brain, lung, and blood vessels, and plays a role in regulation of a potent vasoactive peptide hormone, angiotensin II. ACE2 also acts as anti-inflammatory, and inhibition of ACE2 results in overactivation of inflammatory pathways. Additionally, psychosocial stressors caused by COVID-19 may also contribute to autonomic dysfunction. Autonomic dysfunction is characterized by elevated sympathetic activity and withdrawal of parasympathetic activity and is a common pathophysiological condition in patients with heart disease, hypertension, and diabetes [20].

Hyposmia is a well-known neurological manifestation of COVID-19. However, hyposmia occurred in only 27 (6%) of our patients, likely due to the older age and high rate of dementia in our sample. Hyposmia may be due to infection of olfactory epithelium and trigeminal nerves by SARS-CoV-2 [21,22]. Altered mental status is another commonly documented CNS manifestation of COVID-19 and occurred in 92 (19%) of our patients. Prior research suggests that altered mental status is a particularly lethal manifestation of COVID-19, specifically in older adults presenting to the emergency department, in most cases causing acute on chronic neurocognitive dysfunction strongly influenced by systemic inflammation and hypoxic-ischemic mechanisms [5].

Our study identified myalgia and headache as the most common neurological manifestations. Limited information exists regarding the mechanisms and timing of headache in patients with COVID-19. Direct viral invasion of the nervous system as well as the cytokine release syndrome can cause headache. Headache may be due to infection of nasal cavity trigeminal nerve endings and/or endothelial cells in the trigeminovascular system, and/or irritation of trigeminal nerve endings due to increased proinflammatory cytokines [23].

The association of comorbid conditions with neurological manifestations has been reported previously; however, the specific association of comorbid conditions with CNS manifestations, PNS manifestations, and combined CNS/PNS manifestations was unclear. Our study finds that several comorbid conditions were associated with CNS manifestations, though no comorbidities were associated with PNS manifestations. It is unclear whether cardiac disease, hypertension, dementia, and/or COPD made these patients more prone to CNS manifestations or whether systemic inflammation and/or other mechanisms led to CNS involvement.

Study limitations

There are limitations of our study that are worth mentioning. As a retrospective study focused on acute neurological manifestations, we lack information regarding persisting problems and outcomes. Furthermore, we lack data on the results of diagnostic studies that could enhance the results of our findings such as results of serology, electroencephalography, and neuroimaging. We also lack data on patient medications, which could also influence treatment options for COVID-19 manifestations.

## Conclusions

Medical comorbidities and neurological manifestations are common in COVID-19 patients. The most common CNS manifestations in our sample were headache and altered mental status. The most common PNS manifestation was myalgia. Comorbid conditions such as cardiac disease, dementia, hypertension, and COPD were more prevalent in patients with CNS manifestations. Future research may further investigate CNS and PNS manifestations and their relationships with laboratory studies, electroencephalography, neuroimaging, medications, and patient outcomes.

Our study provides further evidence of neurological involvement in COVID-19. To our knowledge, there are few studies specifically analyzing the association between medical comorbidities and CNS or PNS manifestations in COVID-19. Providers should be vigilant about the possible emergence of CNS manifestations in COVID-19 patients with cardiac disease, hypertension, dementia, and COPD. Proper management of comorbid disease conditions in patients with COVID-19 may minimize CNS manifestations leading to improved outcomes.
